# The Effects of Bond–Slip Laws on the Debonding Failure and Behavior of Flexural Strengthened RC Slabs in Hybrid FRP Retrofit Systems

**DOI:** 10.3390/ma15217453

**Published:** 2022-10-24

**Authors:** Huy Q. Nguyen, Tri N. M. Nguyen, Do Hyung Lee, Jung J. Kim

**Affiliations:** 1 Department of Civil Engineering, Kyungnam University, Changwon-si 51767, Korea; 2 Campus in Ho Chi Minh City, University of Transport and Communications, No. 450-451 Le Van Viet Street, Tang Nhon Phu A Ward, Thu Duc City, Ho Chi Minh City 700000, Vietnam; 3 Department of Civil, Railroad and Unmanned Systems Engineering, PaiChai University, 155-40 Baejaero, Seo-gu, Daejeon 35345, Korea

**Keywords:** CFRP, bond–slip law, debonding, RC slab, retrofit, strengthen

## Abstract

The hybrid retrofit system using FRP and concrete overlay applied on the top of slabs has proven effective in strengthening and overcoming logistical constraints, compared with conventional strengthening techniques using externally bonded composite materials to the underside of the slabs. Nevertheless, the performance of retrofitted slabs is governed by debonding failure due to the low bond strength between CFRP and concrete overlay. Thus, this study investigates the behavior of flexural strengthened slabs with FRP retrofit systems and the effect of bond–slip laws on debonding failure. Firstly, two full-scale RC slabs with and without a retrofit system were tested in a four-point bending setup as the control specimens. Then, the same retrofitted slab was simulated by utilizing the commercial program ABAQUS. A sensitivity analysis was conducted to consider the influence of bond–slip laws to predict the failure mechanism of the retrofitted slabs based on load–deflection relationships. The results showed that the strengthened slab enhanced the load-carrying capacity by 59%, stiffness by 111%, and toughness by 29%. The initial stiffness of 0.1K_0_ and maximum shear stress of 0.13τ_max_, compared with the corresponding values of Neubauer’s and Rostasy’s bond–slip law, can be used to simulate the global response of the retrofitted slab validated by experiment results.

## 1. Introduction

Strengthening structures with external bonded materials has become one of the popular choices for rehabilitation and upgrading existing reinforced concrete (RC) structures [1,2]. Researchers and engineers are particularly interested in restoring and strengthening partially damaged RC structures to minimize the impact on environmental deterioration and cost savings [3,4,5]. Fiber-reinforced polymer (FRP) materials have been widely used in the field of external reinforcement in recent decades because of their lightweight, high tensile strength, and non-corrosion resistance [6,7,8,9,10,11,12]. New strengthened techniques and design guidelines have emerged for RC structures using FRP composite materials [13,14,15,16].

The conventional application in strengthening RC slab techniques focuses on attaching FRP to their tensile zone to maximize the tensile strength of these composite materials, which have been widely gaining acceptance in practice [17,18,19]. However, the premature delamination of FRP remains the main disadvantage of the strengthening techniques, as it prevents strengthened slabs from reaching their ultimate load-carrying capacity. In general, sectional and debonding failures are two widespread possible failure modes of FRP-strengthened slabs [20,21,22]. In sectional failure, either the failure of compressive concrete or the rupture of FRP can be predicted through strain compatibility at its limiting strain [23]. In debonding failure, it is more difficult to determine due to the complicated failure mechanism and the factors contributing to problems such as concrete cracks and stress concentration at the FRP–concrete interface. Due to this, the behavior of externally bonded FRP in contact with a concrete surface has attracted considerable interest from the civil engineering community [24,25,26]. In addition, obtaining a well-prepared concrete surface for strengthening FRP on the underside of the RC slab can be a more difficult challenge, or it may not be possible due to logistical constraints. 

**Figure 1:**
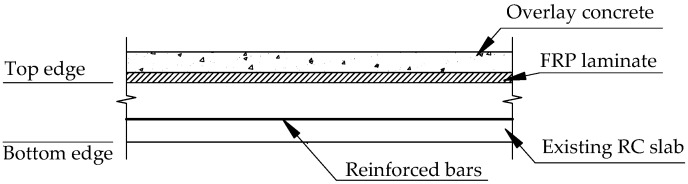
Hybrid FRP retrofit system for positive moment parts of strengthened RC slabs.

Consequently, a hybrid retrofit system consists of carbon fiber reinforced polymer (CFRP) and concrete overlay applied on the top surface of the RC slab to overcome the shortcomings of traditionally strengthened solutions rather than exploit the high tensile strength of CFRP, as shown in Figure 1. Previous studies have demonstrated the effectiveness of the retrofit system by increasing the flexural strength and ductility of existing RC slabs. Nevertheless, the results also revealed that debonding failure between the concrete overlay and CFRP was one of the major causes of slabs losing their ultimate load-carrying capacity. Transferring interfacial stress from the slab to FRP or vice versa via a specialized adhesive layer is more efficient than from FRP to overlay members due to the characteristics of the retrofit system, leading to the premature debonding failure of the FRP/overlay interface [27,28,29]. A sound understanding of the behavior of FRP–concrete interface needs to be developed, with a particular interest in FRP/overlay interface for safe in practical design work.

In response to this need, the effect of bond–slip laws on the debonding failure of flexural strengthened RC slabs with a hybrid FRP retrofit system is investigated. To the best of our knowledge, previous studies were limited in evaluating the behavior of the externally bonded FRP on a concrete surface without considering cases of CFRP located between two concrete layers of the strengthened slab with a retrofit system [30,31,32,33,34,35,36,37]. There was still considerable uncertainty and difficulty in determining the behavior of CFRP-to-concrete joints due to their intrinsic bond. Insight into an understanding of the bond–slip law between concrete and CFRP using a cohesive approach is a suitable solution for predicting strengthened structural responses [36,38]. Shear stress at the FRP–concrete interface of cohesive elements, in terms of magnitude and distribution, could be a believable explanation for debonding failure [39,40]. It can be difficult to precisely predict the variations in stresses in concrete structures, whereas the load–deflection curve is far less sensitive to crack locations and sizes. The finite element method (FEM) can reasonably predict the interfacial stresses and the delamination load corresponding to deflection more economically than laboratory tests [41]. A finite element (FE) model of the FRP flexural-strengthened slab can provide a comprehensive understanding of the various bond–slip parameters associated with the CFRP/concrete interface and be applicable in developing robust predictive equations for practice designs [42]. By combining FEM through ABAQUS software and the experimental program, the iterative adjustment of the bond–slip parameters can be easily carried out to simulate the experimental load–displacement curve and failure modes, resulting in reduced experiment time and cost [43]. 

In this study, the behavior of a strengthened RC slab with a hybrid FRP retrofit system is described with particular attention to debonding failure. The effectiveness of the hybrid retrofit system in improving the flexural carrying capacity, stiffness, and toughness is investigated and compared with the full-scale experimental results. A three-dimensional FE model is developed with various bond–slip models for evaluating their accuracy by comparing numerical predictions with experimental measurements. Numerical models are also analyzed for sensitivity to the bond–slip parameters to assess their effects on the debonding failure. Following the validation of the model, the various parameters that strongly influence the behavior of retrofitted slabs are identified and discussed.

## 2. FRP Hybrid Retrofit System

### 2.1. Retrofitting Mechanism

**Figure 2:**
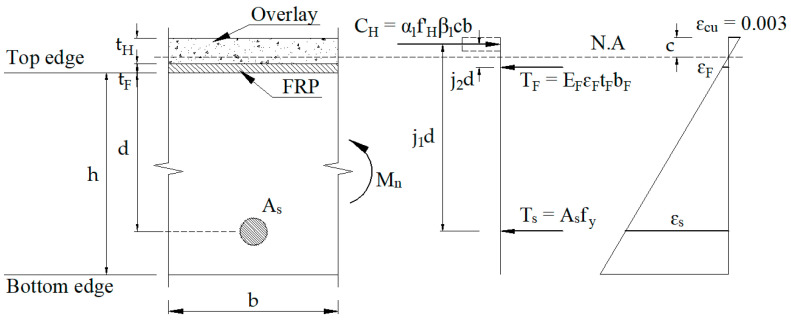
Calculating mechanism for slab’s positive moment sections with a proposed system.

In some cases, strengthening the underside of the reinforced concrete slabs may not be possible due to logistical constraints and hindrances by other utilities. A hybrid retrofit system combining the tensile strength of FRP and compressive strength of the concrete overlay was applied on top of the existing RC slabs to improve their strength and ductility, as shown in Figure 1. The overlay thickness of the retrofit system has a critical role in pulling the neutral axis toward the overlay zone, and FRP holds tension at failure. For this case, the retrofitting mechanism for the hybrid FRP system was estimated as recommended by ACI 440.2R [44], as shown in Figure 2. Assume that the ultimate concrete strain (ε_cu_) is 0.003, and steel yields at yield stress of f_y_. Without considering the tension force of concrete, the force equilibrium is calculated as follows:

(1) $$C_{H}{=T\;}_{s}{+T}_{F}$$

These internal forces are computed as follows:

(2) $$C_{H}=\alpha_{1}f_{H}^{\prime }\beta_{1}\mathrm{cb},\;T_{s}=A_{s}f_{y},\;T_{F}=E_{F}\varepsilon_{F}t_{F}b_{F}$$

An evaluation of strain compatibility conditions can determine FRP strain at strength limit (ε_F_) as follows:

(3) $$\varepsilon_{F}=\left[\frac{\left(t_{H}+t_{F}/2\right)-c}{c}\right]\varepsilon_{\mathrm{cu}}$$

The moment capacity equilibrium condition can be defined as follows:

(4) $$M_{n}{=T}_{s}{(j}_{1}{d)+T}_{F}{(j}_{2}d)$$

### 2.2. Experimental Program

**Figure 3:**
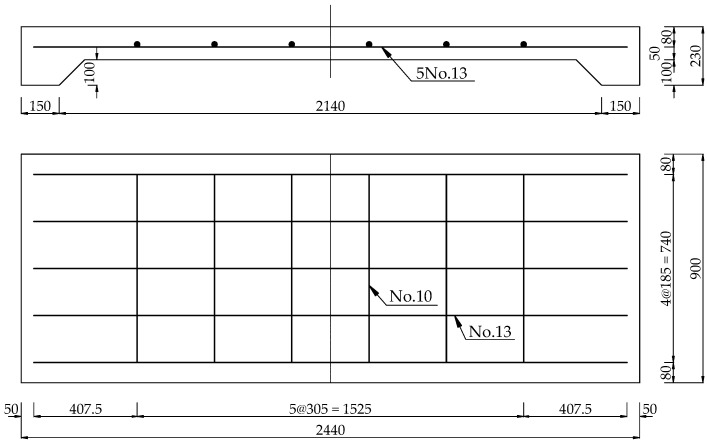
Reference slab reinforcement details.

The reference slab had a 2440 mm total length, 2290 mm clear span, and 130 × 900 mm^2^ cross-section. The tensile reinforcement was spaced at 185 mm spacing with five No.13 bars (*ϕ*12.7 mm) at the bottom of the RC slab. Transverse steel consisted of six No.10 bars (*ϕ*9.5 mm) spaced at 305 mm center on the center, as shown in Figure 3. The yield stress of the reinforcement used for the RC slab was 400 MPa. The concrete employed for the RC slab had a compressive strength of 27 MPa after 28 days. Table 1 provides the mechanical properties and dimensions of the reference slab. 

A hybrid retrofit system was constructed utilizing a combination of CFRP wet layup laminate with a concrete overlay, as shown in Figure 1. The compressive strength of the concrete overlay was 50 MPa at 28-day age. CFRP had a strength of 600 MPa and an elastic modulus of 40 GPa. The mechanical properties and dimensions used in the FRP retrofit system are given in Table 2. 

**Figure 4:**
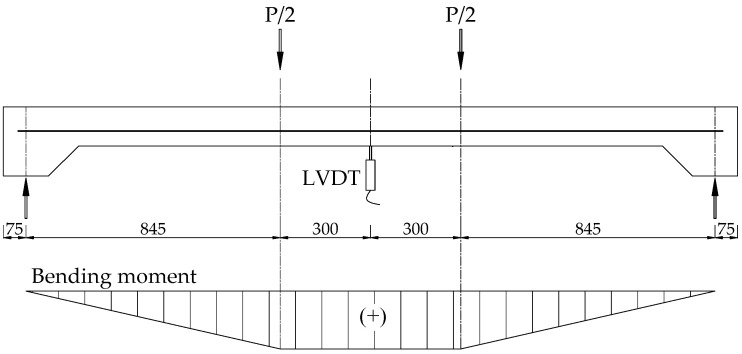
Four-point bending setup for slab and the corresponding moment of the applied load.

Two full-scale RC slabs with and without a hybrid retrofit system were tested in a four-point bending setup with two concentrated loads, as shown in Figure 4. Before measuring the deflection data, the LVDTs were reset to remove self-weight deflection. The load cell capacity applied in the tests was 5000 kN. The applied load and deflection at the mid-span section data were recorded during the experimental procedure.

### 2.3. Theoretical Analysis

The experimental data were checked with the predicted analytical moment capacity and deflection at the mid-span due to the applied load of the reference and strengthened RC slabs, as recommended by ACI 440.2R [44] and ACI 318M [45]. Based on the structure, a self-weighted equivalent distributed load is computed as follows:

(5) $${w=\gamma }_{c}{b(h+t}_{H}{)+\gamma }_{F}b_{F}t_{F}$$

The distributed load due to the self-weight of the reference slab and strengthened slab is determined as follows: W_c_ = 2.81 N/mm and W_s_ = 3.46 N/mm.

The prediction of the load-carrying capacity corresponding to deflection at mid-span due to the applied load for the reference RC slab is given in Table 3.

The RC slab and CFRP laminate were assumed to be in perfect bond until the load-carrying capacity was reached. The prediction of the load-carrying capacity corresponding to deflection at mid-span for the CFRP-retrofitted slab due to the applied load is shown in Table 4. 

## 3. Finite Element Modeling

### 3.1. Finite Element Mesh

**Figure 5:**
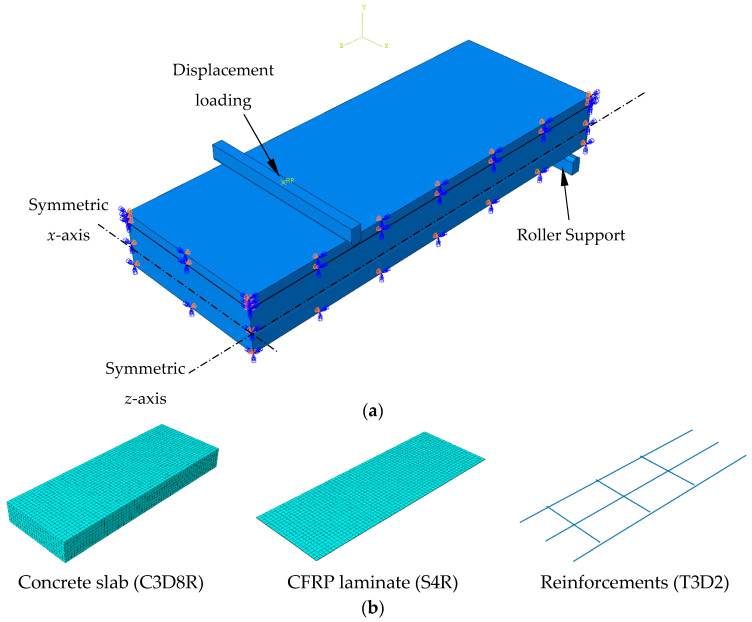
CFRP retrofitted slab (**a**) modeling and boundary conditions; (**b**) element types of the FEM model.

The simulations were conducted using one-quarter of the control and strengthened slab specimens, based on specimen symmetry. The symmetric pane was simulated for the *x*- and *z*-axis by restricting translation in directions 1 and 3. The concrete slab and concrete overlay with reduced integration were constructed with eight-node solid elements (C3D8R). Two-node linear truss elements (T3D2) and four-node shell elements (S4R) were used to model the reinforcements and CFRP laminate, respectively. A mesh size of 20 mm was suggested based on preliminary mesh refinement studies, ranging from 10 mm to 25 mm, for balancing accuracy and computational cost. The embedded function in ABAQUS/CAE 2022 was implemented to simulate concrete–steel bonding. The models were studied using static analysis in ABAQUS/standard. The models under monotonic displacement loads surveyed the behavior of a slab subjected to a four-point bending test. The steel bar was discretized as a rigid part to prevent manufactured stress concentrations under the loading points, as shown in Figure 5.

### 3.2. Concrete

A model of concrete plastic damage was used with a failure mechanism of compressive crushing and tensile cracking. The stress–strain curve for concrete under uniaxial compression and tension was calculated by using the CEB–FIP model [46]. Compressive strength was used to estimate the tensile strength (f_ct_) and elastic modulus (E_c_) of concrete, as recommended by ACI 318M [45].

(6) $$f_{\mathrm{ct}}=0.62\sqrt{f_{c}^{\prime }}$$

(7) $$E_{c}=4700\sqrt{f_{c}^{\prime }}\;$$

Fracture energy (G_cr_) is an inelastic parameter associated with the softening part of the curve. In mode I, fracture energy is defined as the area under the softening curve.

(8) $$G_{\mathrm{cr}}=G_{f0}{\left(\frac{\;f_{c}^{\prime }}{10}\right)}^{0.7}$$

**Figure 6:**
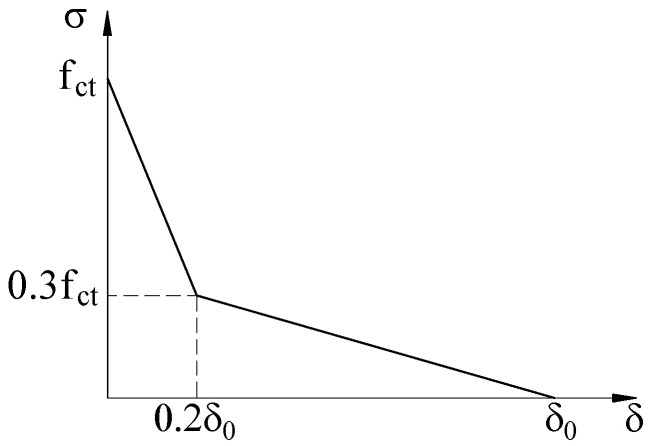
Concrete softening curve under uniaxial tension.

Figure 6 illustrates the concrete softening curve under uniaxial tension. A linear relationship between the tension damage and crack opening (δ) is assumed to identify tensile damage. The tension damage variable ranges from zero (undamaged material) to one (total loss of strength).

Under uniaxial compression, a linear response is observed until reaching the initial value (σ_c0_). In the plastic regime, the response is generally characterized by stress hardening, followed by strain softening beyond the ultimate compressive stress [47].

(9) $$\sigma_{c}=\frac{E_{c}\varepsilon_{c}}{1+\left(R+R_{E}-2\right)\left(\varepsilon_{c}{/\varepsilon }_{0}\right)-\left(2R-1\right){\left(\varepsilon_{c}{/\varepsilon }_{0}\right)}^{2}+R{\left(\varepsilon_{c}{/\varepsilon }_{0}\right)}^{3}}$$

where $R=\left[R_{E}\left(R_{\sigma }-1\right)/{\left(R_{\varepsilon }-1\right)}^{2}\right]-{1/R}_{\varepsilon }$; $R_{E}=E_{c}{/E}_{0}$; $E_{0}=\;f_{c}^{\prime }{/\varepsilon }_{0}$; $\varepsilon_{0}=0.0025$; $R_{E}=R_{\sigma }=4\;$ [48].

### 3.3. Reinforced Steel

It is assumed that the reinforcement transmits force axially, and the most common perfectly linear–elastic model for reinforcement was used. Steel’s Poisson’s ratio is 0.3, and the other mechanical properties are listed in Table 2.

### 3.4. CFRP

For strengthening, CFRP laminate is supposed to be linear and exert isotropic behavior. CFRP failure criteria were not considered for this study because tensile stress in CFRP is too low, compared with its ultimate strength, and therefore CFRP laminate hardly ruptures for these types of structures. The material properties of CFRP, including its thickness, elastic modulus, and ultimate strength, as shown in Table 2, are provided by manufacturers.

### 3.5. FRP-to-Concrete Interface Model

**Figure 7:**
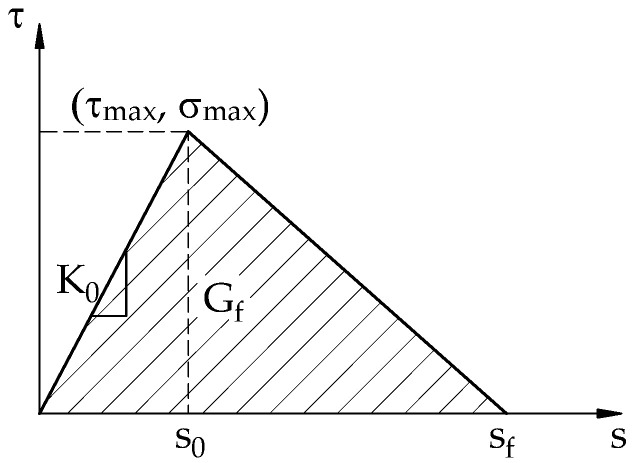
Bilinear traction–separation response.

The FRP-to-concrete interface was modeled using cohesive zones. In this study, a bilinear traction–separation model was employed to depict the bonding characteristics of linear adhesive at the interface, as shown in Figure 7 [49]. 

The initiation and evolution of damage in the available traction–separation model were initially assumed to be a linear–elastic behavior [50,51], which can be written in a matrix form as follows:

(10) $$\left\{\begin{array}{l} \tau_{n}\\ \tau_{s}\\ \tau_{t} \end{array}\right\}=\left[\begin{array}{ccc} K_{\mathrm{nn}} & 0 & 0\\ 0 & K_{\mathrm{ss}} & 0\\ 0 & 0 & K_{\mathrm{tt}} \end{array}\right]\left\{\begin{array}{l} \sigma_{n}\\ \sigma_{s}\\ \sigma_{t} \end{array}\right\}$$

For specifying the damage initiation, quadratic nominal stress and maximum nominal stress between the CFRP and concrete interface were employed. The damage criterion for quadratic stress may be depicted as follows:

(11) $${\left(\frac{\langle \tau_{n}\rangle }{\sigma_{\max}}\right)}^{2}+{\left(\frac{\tau_{s}}{\tau_{\max}}\right)}^{2}+{\left(\frac{\tau_{t}}{\tau_{\max}}\right)}^{2}=1$$

The damage criterion for maximum stress can be described as follows:

(12) $$\max\left(\frac{\langle \tau_{n}\rangle }{\sigma_{\max}},\frac{\tau_{s}}{\tau_{\max}},\frac{\tau_{t}}{\tau_{\max}}\right)=1$$

For specifying the damage evolution, the influence of power law and the Benzeggagh–Kenane (BK) fracture criteria on the structure behavior was further evaluated [52]. The power law criterion may be given by:

(13) $${\left(\frac{G_{n}}{G_{n}^{f}}\right)}^{\eta }+{\left(\frac{G_{s}}{G_{s}^{f}}\right)}^{\eta }+{\left(\frac{G_{t}}{G_{t}^{f}}\right)}^{\eta }=1$$

The BK fracture criterion may be defined as follows:

(14) $$G_{n}^{f}+(G_{s}^{f}-G_{n}^{f}){\left(\frac{G_{s}+G_{t}}{G_{n}+G_{s}}\right)}^{\eta }=G_{f}$$

In this study, the quantities $G_{n}^{f},{\;G}_{s}^{f},{\;\mathrm{and}\;G}_{t}^{f}$ were assumed to be equal. For FE modeling, first, a perfect FRP-to-concrete bond was assumed to reflect the response of the flexural strengthened RC slab using the ABAQUS program. Then, the interaction behavior of the FRP-to-concrete bond in the strengthened slab applied to the retrofit system was evaluated using the existing constitutive models, as shown in Table 5 [31,32,33,34,35,36].

It is easily conceptualized that the traction–separation law, as described in Figure 7, is primarily governed by three parameters, namely initial stiffness (K_0_), maximum shear stress (τ_max_), and fracture energy (G_f_) [53]. Based on the mechanical properties of the experimental materials, the bond–slip models evaluated in this study were introduced with the three characteristic parameters of the traction–separation law, as shown in Table 6.

### 3.6. Analysis Procedure and Flowchart

**Figure 8:**
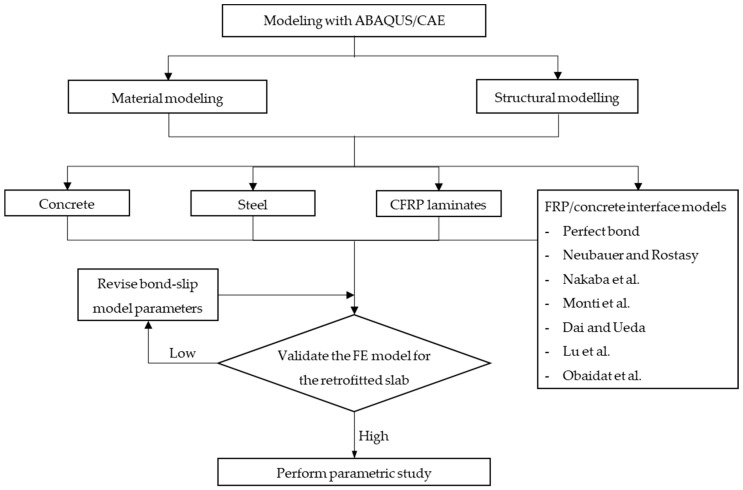
FE model analysis flowchart.

A three-dimensional FE model using ABAQUS/CAE 2022 software was performed to simulate the behavior of retrofitted slabs. In the initial phase, an accurate FE model of the reference slab was assessed by comparison with the experimental results. In the second phase, a retrofitted slab model was developed based on the reference slab model, with the addition of FRP laminate and concrete overlay. The well-known bond–slip models were used to simulate the FRP-to-concrete interface to accurately simulate the behavior of the retrofitted slab validated by experiment results. If the available bond–slip models were not appropriate, the bond–slip parameters were revised to show good agreement with the experimental data. A flowchart of the analysis procedure for the FE model is shown in Figure 8.

## 4. Results and Discussion

### 4.1. Experimental Analysis

**Figure 9:**
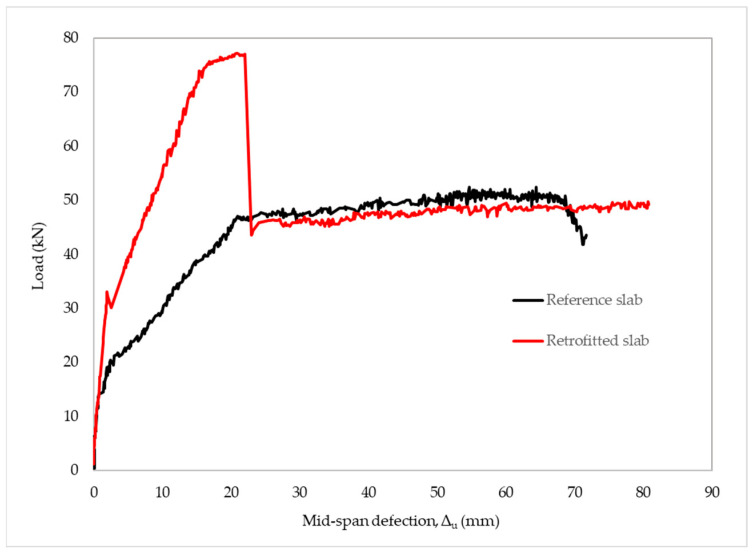
Load–deflection relationship for reference and retrofitted RC slabs.

The experimental data from the reference slab showed a good agreement with those of the theoretical analysis. The RC slab behaved linearly and elastically until the first crack appeared at 17 kN of the applied load. The observed result revealed a higher load-carrying capacity of 43.5 kN than the theoretical prediction of 39.9 kN. The ultimate loading (50.8 kN) was 27.3% greater than the predicted load. The linearly elastic–perfectly plastic behavior of the steel can be the reason for the lower strength of the prediction because strain-hardened steel shows higher tensile strength in practice. The mid-span deflection reached a maximum value of 72 mm, as shown in Figure 9. The slope of the load–deflection curve within the elastic limits depicts the stiffness (K) of the slab. The stiffness of the RC slab was determined as 10.1 kN/mm. Calculating the toughness of a structure can be accomplished by integrating the load–deflection curve. Accordingly, the reference slab had a toughness of 3129.2 kNmm.

**Figure 10:**
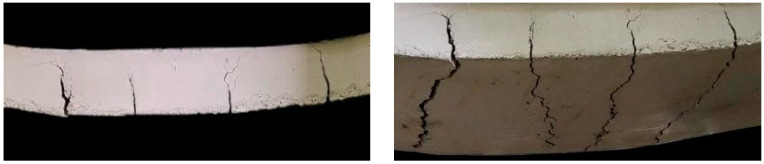
The cracks at the mid-span of the slab: (**a**) front view; (**b**) bottom view.

For the retrofit slab, the cracks emerged and expanded most strongly at the mid-span zone—between the loading points, as shown in Figure 10. The retrofitted slab showed a load-carrying capacity of 69.2 kN at 14 mm mid-span deflection. This was 9% higher than the predicted load of 63 kN. The structure lost carrying capacity due to the micro-buckling of CFRP laminate at the applied load of 32 kN. Then, the applied load continued to increase until CFRP laminate was delaminated at 76.9 kN, corresponding to 22 mm of the mid-span deflection. After delamination, the global response of the concrete slab was equivalent to that of the reference concrete slab. Its failure load and mid-span deflection were 49.2 kN and 81 mm, respectively, as shown in Figure 9. It is possible to define the stiffness of the retrofitted slab at 21.3 kN/mm, which was 2.11 times that of the reference slab. The retrofitted slab had a toughness of 4034.1 kNmm, 1.29 times greater than the reference slab. Compared with theoretical analysis, Table 7 summarizes the experimental results for load-carrying capacity corresponding to the mid-span deflection of the slabs.

### 4.2. Numerical Analysis

**Figure 11:**
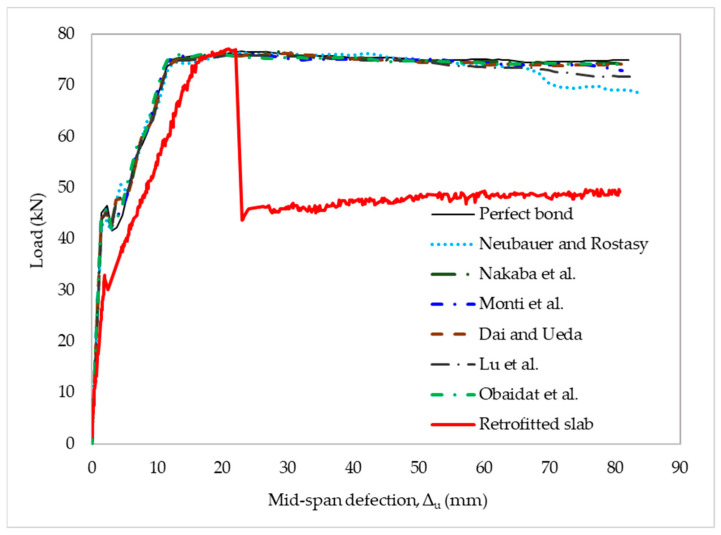
Experimental and FEM models of load–deflection curves for retrofitted slabs.

In this part of the study, the FE model of the retrofitted slab was used to examine the failure mode and delamination load after obtaining a good agreement between the FE model of the reference slab and the experimental results using the commercial ABAQUS program. A perfect bond model and well-known bond–slip models were applied to simulate the slab behavior through the load–deflection relationship. The material properties of the experimental structure were used to calculate the input parameters for the bond–slip models, as shown in Table 6. The FE models were employed to evaluate the suitability of bond–slip laws based on the observed results. Most existing bond–slip models can predict the load-carrying capacity of the retrofitted slab when errors in the prediction of FE models are less than 7%. Nevertheless, the bond–slip models could not accurately predict the mid-span deflection corresponding to the load-carrying capacity and stiffness, with errors higher than 17% and 32%, respectively. The behavior of the well-known bond–slip models also showed a perfect bond between CFRP and concrete, even though delamination failure was observed in the experiment, as shown in Figure 11. These discrepancies may have resulted from the improper estimations of the bond–slip parameters involving fracture energy, the initial stiffness of the interface elements, maximum interfacial stress, damage initiation criteria, and mixed-mode failure criteria. The FEM results of the load-carrying capacity corresponding to the mid-span deflection and stiffness within the elastic limits of the strengthened slab, compared with its experimental counterpart, are summarized in Table 8.

### 4.3. Bond–Slip Analysis

**Figure 12:**
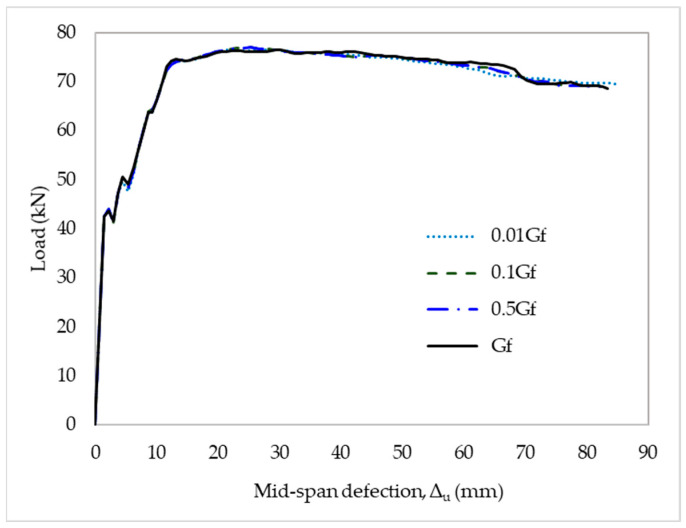
Sensitivity to fracture energy of CFRP-to-concrete interface.

The existing bond–slip models tended to overestimate the bond strength of the CFRP-to-concrete interface. In this part of the study, a sensitivity analysis of the bond–slip input parameters was performed to compare its results with the structural behavior obtained from the experimental data. The bond–slip model of Neubauer and Rostasy was used to illustrate the numerical results from the strengthened slabs. Initially, the CFRP and concrete bonding interface was assumed to fail using quadratic traction. A cohesive property coefficient of η = 1 was applied to depict the influence of the opening and sliding failure modes on fracture energy. The authors investigated the sensitivity of the results in the interfacial fracture energy between CFRP and concrete. For this case, a change was made from 0.01G_f_ to G_f_ for the interfacial fracture energy. There was no evidence that G_f_ had a notable effect on the numerical results, as shown in Figure 12.

**Figure 13:**
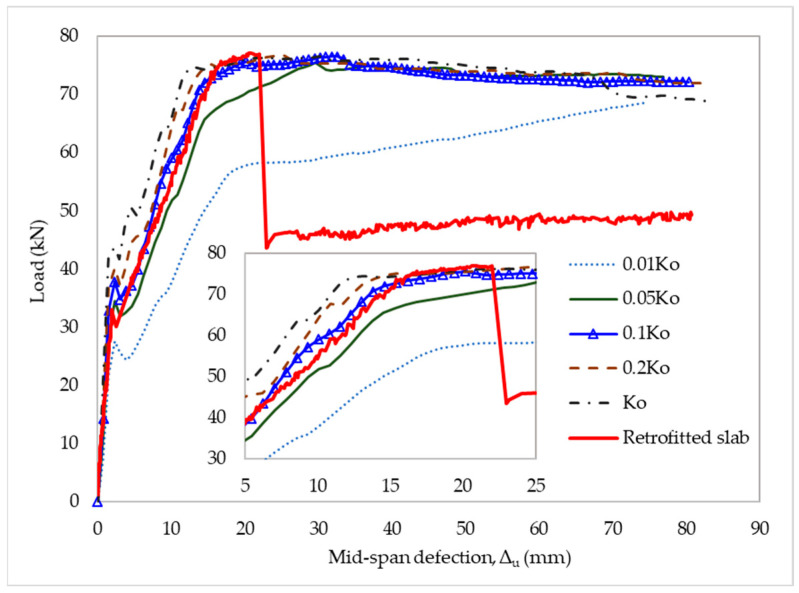
Sensitivity to initial stiffness of interface elements.

The initial stiffness of the interface elements was changed from 0.01K_0_ to K_0_ to evaluate its effect. It had a substantial influence on the load–deflection curve’s slope of the structure. Based on the simulated results, the stiffness of the retrofitted slab decreased with decreasing the initial stiffness but was not proportional. With the initial stiffness of 0.1K_0_, the global response of the slab within the elastic limits could match the experimental results, as shown in Figure 13. However, the constitutive model exhibited a perfect bond of CFRP-to-concrete interface. 

**Figure 14:**
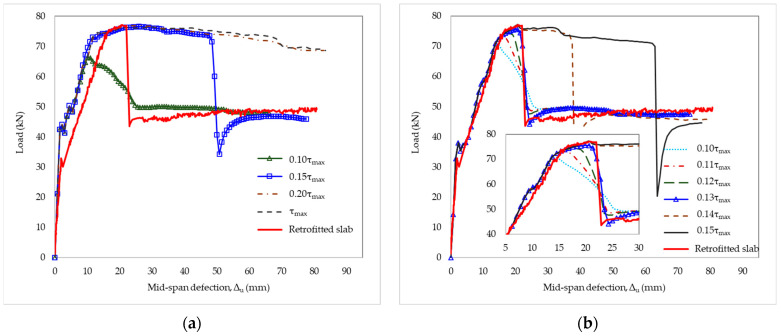
Sensitive to the maximum shear stress (**a**) initial stiffness of K_0_; (**b**) initial stiffness of 0.1K_0_.

As mentioned above, the low bond strength between the concrete overlay and CFRP was the main reason for the delamination failure of the retrofit slab. It was possible to obtain better explanations for the differences between the simulated and experimental results by modifying the maximum shear stress. Therefore, the maximum interfacial stress of the concrete overlay/CFRP was further investigated using sensitivity analysis. An appropriate value for this parameter was crucial for the accurate prediction of CFRP debonding failures. As shown in Figure 14a, the maximum shear stress was increased from 0.1τ_max_ to τ_max_. The shear stress values had a considerable impact on the debonding failure. The delamination load of the retrofitted slab was sensitive to changes in the ranges of maximum shear stress from 0.1τ_max_ to 0.15τ_max_, whereas a perfect bond was shown for values greater than or equal to 0.2τ_max_. It could also be easily seen that Neubauer’s and Rostasy’s bond–slip law, along with other equivalent models, overestimated the ultimate shear strength of the concrete overlay–CFRP interface. 

**Figure 15:**
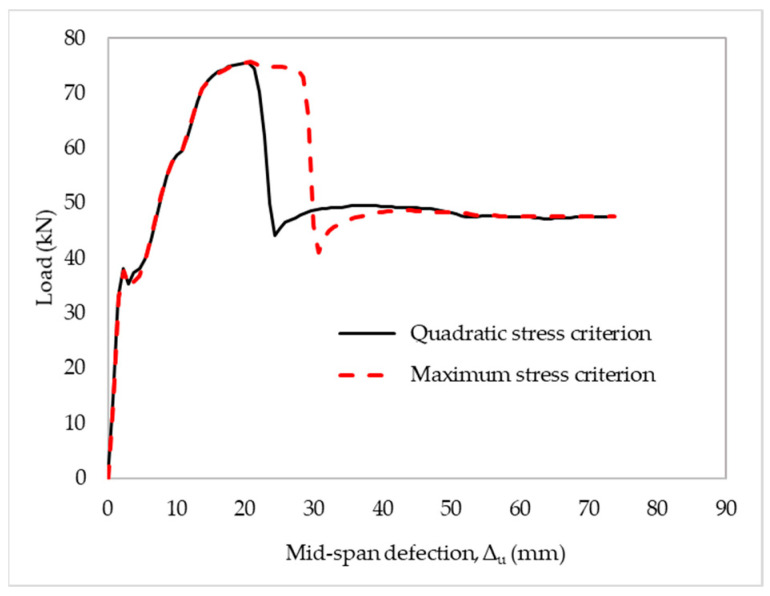
The effect of strengthened slabs on damage initiation criteria.

According to the simulations, the delamination loads were 66.2 kN and 74 kN, corresponding to 10.1 mm and 48 mm of the mid-span deflection, respectively, occurring with the maximum shear stresses of 0.1τ_max_ and 0.15τ_max_, respectively. The numerical results also indicated that the debonding failure emerged earlier, at 0.1τ_max_ and later at 0.15τ_max_, compared with the experimental results. Moreover, the overestimation of the initial stiffness should also be highlighted in this case. The maximum shear stress ranged from 0.1τ_max_ to 0.15τ_max_, combined with the initial stiffness of 0.1K_0_; thus, the case was further evaluated. The delamination load and mid-span deflection of the retrofitted slab increased with the increase in the maximum shear stress but was not proportional; however, there were no notable differences in the delamination load after the yield in steel. The global response of the strengthened slab was displayed as a function of the shear stress values. As a result, it was possible to reasonably simulate the behavior of the retrofitted slab and compare it with its experimental counterpart with the initial stiffness of 0.1K_0_ and maximum shear stress of 0.13τ_max_, as shown in Figure 14b. In this part of the study, we analyzed the damage initiation criteria as the quadratic and maximum nominal stresses based on the proposed results mentioned above. The quadratic stress criterion is defined by accounting for the effect of traction components, while the maximum stress criterion is established by comparing the traction components to their respective allowable values [54]. Despite having similar responses until the yield in steel, the quadratic and maximum nominal stress criteria exhibited different mid-span deflections at debonding failures of 22 mm and 28 mm, respectively, as shown in Figure 15. Poor results were obtained using the maximum stress criterion for predicting delamination in the retrofitted slabs. There was a significant interaction between stresses for decohesion elements dealing with mixed-mode delamination onset and propagation, as mentioned by Cui et al. [55]. Debonding behavior can emerge before any of the involved traction components reach their respective allowable limits. An accurate prediction of delamination failure requires taking into account the interaction. Thus, the damage initiation criterion applying the quadratic nominal stress was suggested in this case.

**Figure 16:**
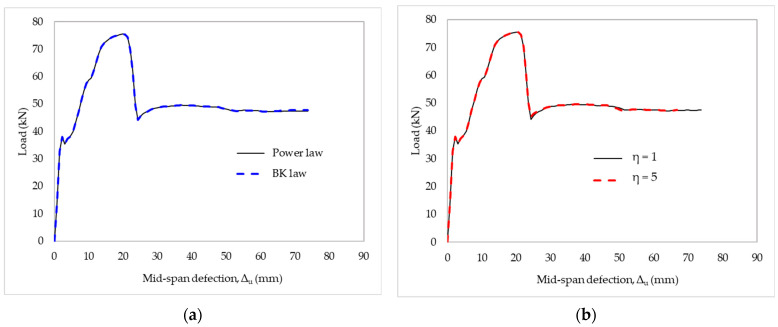
The effects of strengthened slabs on damage evolution: the criteria of (**a**) mixed-mode failure and (**b**) cohesive parameter (η).

Here, we describe the effects of damage evolution based on energy. The load–deflection relationship was derived from the power law or BK law, as shown in Figure 16a. Likewise, a study of the cohesive coefficient sensitivity of FE results was also conducted, the result of which is shown in Figure 16b. Nevertheless, these criteria had no effect on the delamination load or global response of the strengthened RC slabs.

In brief, these results were consistent with the previous literature regarding the influential factors on debonding failure, which is significantly affected by the maximum interfacial stress [40,56,57] but insensitive to changes in fracture energy and mixed-mode failure criteria [53]. However, disagreement can also be observed once the global response of the strengthened slab with a hybrid retrofit system was found to be impacted by changing the criteria of the stiffness of interface elements and damage initiation. Developing an appropriate bond–slip model will require further investigation to determine the precise values for notable influential factors on debonding failure.

## 5. Conclusions

In this paper, a hybrid retrofit system considering the difficulty of accessing and installing CFRP laminates to the underside of RC slabs to enhance the flexural carrying capacity, stiffness, and toughness of the existing RC slabs was proposed. The proposed retrofitting mechanism proved suitable through a good agreement between the experimental results and theoretical analyses at the mid-span section. The global response and the effect of the bond–slip law in predicting the debonding failure of the retrofitted RC slabs were presented. The FE model using a sensitivity analysis based on the load–deflection relationship was performed to evaluate the parameters influencing the interfacial behavior between CFRP and concrete. Based on the obtained results, the following conclusions could be drawn as follows: 

The efficiency of the retrofit system was verified through the experimental test of the strengthened slab in the positive moment section. The retrofit system enhanced the load-carrying capacity of the slab by 59%, stiffness by 111%, and toughness by 29%.

The neutral axis of the retrofitted slab was located within the overlay, and CFRP held tension at the mid-span section at the ultimate failure state.

The bond–slip models overestimated the criteria regarding the damage initiation between CFRP and the concrete overlay. The numerical analysis results with the initial stiffness of 0.1K_0_ and maximum shear stress of 0.13τ_max_ were compared with the corresponding values of Neubauer’s and Rostasy’s bond–slip law and showed a good agreement with the experimental data. 

The stiffness of structures is notably impacted by the initial stiffness of the interface elements, while the delamination load and failure load can be decided by the maximum interfacial stress. Quadratic nominal stress is recommended as a criterion for damage initiation based on the considerable interaction between stresses.

The global response of the retrofitted slab was not sensitive to changes in the interfacial fracture energy and damage evolution regarding the mix-mode failure criteria and the cohesive coefficient of the interface.

Our study contributes to the evaluation of the impact of bond–slip parameters on the behavior of retrofitted slabs. Nonetheless, further research into an appropriate bond–slip model for retrofitted RC structures should consider the influence of factors regarding materials properties and geometric dimensions.

## Figures and Tables

**Table 1:** Mechanical properties and dimensions of reference slab.

h (mm)	b (mm)	A_s_ (mm^2^)	d (mm)	f′_c_ (MPa)	γ_c_ (kg/m^3^)	f_y_ (MPa)	E_s_ (GPa)
130	900	632.5	80	27	2400	400	200

**Table 2:** Mechanical properties and dimensions for retrofit system.

Overlay		CFRP				Epoxy Resin		
t_H_ (mm)	f′_H_ (MPa)	t_F_ (mm)	f_tF_ (MPa)	γ_F_ (kg/m^3^)	E_F_ (GPa)	t_a_ (mm)	E_a_ (MPa)	G_a_ (MPa)
30	50	0.5	600	1200	40	1.5	3000	2100

**Table 3:** Analysis of the reference RC slab.

Calculation	Reference Slab
Compressive concrete block depth $a=\frac{A_{s}f_{y}}{\alpha_{1}f_{c}^{\prime }b}$	$a=\frac{632.5(400)}{0.85(27)(900)}=12.25\;\mathrm{mm}$
Moment capacity $M_{n,P}=\left(d-\frac{a}{2}\right)A_{s}f_{y}$	$M_{n,P}=\left(80-\frac{12.25}{2}\right)(632.5)(400)=18.69\times 10^{6}\;\mathrm{Nmm}=18.69\;\mathrm{kNm}$
Moment due to self-weight $M_{c}=w_{c}l^{2}/8$	$M_{c}=2.81{\left(2290\right)}^{2}/8=1.84\times 10^{6}\;\mathrm{Nmm}$
Mid-span section’s remaining capacity for carrying additional load $P_{u,\mathrm{theo}.}=\frac{2(M_{n,P}-M_{c})}{x}$	$P_{u,\mathrm{theo}.}=\frac{2(18.69-1.84)}{845/1000}=39.9\;\mathrm{kN}$
Deflection at yield point steel due to the applied load $\Delta_{u,\mathrm{theo}.}=\frac{\left(3l^{2}-4x^{2}\right)}{24}\frac{\left(M_{n,P}-M_{c}\right)}{E_{c}I_{e}}$ $I_{e}=\frac{I_{\mathrm{cr}}}{1-{\left(\frac{(2/3)M_{\mathrm{cr}}}{M_{n,P}}\right)}^{2}\left(1-\frac{I_{\mathrm{cr}}}{I_{g}}\right)}$ $M_{\mathrm{cr}}=f_{r}I_{g}{/y}_{t}$	$\Delta_{u,\mathrm{theo}.}=\frac{3(2290^{2})-4(845^{2})}{24}\frac{\left(18.69\times 10^{6}-1.84\times 10^{6}\right)}{24,422(22\times 10^{6})}=16.8\;\mathrm{mm}$ $I_{e}=\frac{20.4\times 10^{6}}{1-{\left(\frac{\left(2/3)(8.17\times 10^{6}\right)}{18.69\times 10^{6}}\right)}^{2}\left(1-\frac{20.4\times 10^{6}}{164.8\times 10^{6}}\right)}=22\times 10^{6}{\;\mathrm{mm}}^{4}$ $M_{\mathrm{cr}}=3.22(164.8\times 10^{6})/65=8.17\times 10^{6}\;\mathrm{Nmm}=8.17\;\mathrm{kNm}$

**Table 4:** Analysis of the retrofitted RC slab.

Calculation	Retrofitted Slab
Compressive concrete block depth $J_{1}=\alpha_{1}f_{H}^{\prime }b$ $J_{2}=t_{F}b_{F}E_{F}\varepsilon_{c}-A_{s}f_{y}$ $J_{3}=-\beta_{1}t_{F}b_{F}E_{F}\varepsilon_{c}{(t}_{H}+t_{F}/2)$ $a=\frac{-J_{2}+\sqrt{J_{2}^{2}-{4J}_{1}J_{3}}}{2J_{1}}$	J_1_ = 38,250 N/mm J_2_ = −199,000 N J_3_ = −1,131,782 Nmm a = 8.63 mm
Moment capacity $\begin{array}{l} M_{n,P}=\left(d+t_{H}+t_{F}-\frac{a}{2}\right)A_{s}f_{y}\\ +\left(t_{H}+\frac{t_{F}-a}{2}\right)t_{F}b_{F}E_{F}\varepsilon_{c}\left(\frac{\beta_{1}\left(t_{H}+t_{F}/2\right)}{a}-1\right) \end{array}$	M_n,P_ = 28.87 × 10^6^ Nmm = 28.87 kNm
Moment due to self-weight $M_{s}=w_{s}l^{2}/8$	M_s_ = 2.27 × 10^6^ Nmm = 2.27 kNm
Mid-span section’s remaining capacity for carrying additional load $P_{u,\mathrm{theo}.}=\frac{2(M_{n,P}-M_{s})}{x}$	P_u,theo._ = 63 kN
Deflection at yield point steel due to the applied load $\Delta_{u,\mathrm{theo}.}=\frac{\left(3l^{2}-{4x}^{2}\right)}{24}\frac{\left(M_{n,P}-M_{s}\right)}{E_{c}I_{e}}$	Δ_u,theo._ = 12.3 mm I_e_ = 35 × 10^6^ mm^4^ M_cr_ = 12.5 kNm

**Table 5:** Existing bond–slip models.

Bond–Slip Model	
Neubauer and Rostasy [31]	$\tau =\left\{ \begin{array}{l} \tau_{\max}\left(\frac{s}{s_{0}}\right)\;\;\;\;\;\mathrm{if}\;s\leq s_{0}\\ 0\;\mathrm{if}\;s>s_{0} \end{array}\right.,\;\tau_{\max}=1.8\beta_{w}f_{\mathrm{ct}},{\;s}_{0}=0.202,\beta_{w}=\sqrt{1.125\frac{2-b_{f}/b}{1+b_{f}/400}}$
Nakaba et al. [32]	$\tau =\tau_{\max}\frac{s}{s_{0}}\frac{3}{2+{\left({s/s}_{0}\right)}^{3}},\;\tau_{\max}=3.5f_{c}^{0.19},{\;s}_{0}=0.065$
Monti et al. [33]	$\tau =\left\{ \begin{array}{l} \tau_{\max}\left(\frac{s}{s_{0}}\right)\;\mathrm{if}\;s\leq s_{0}\\ \tau_{\max}\left(\frac{s_{f}-s}{s_{f}-s_{0}}\right)\;\mathrm{if}\;s>s_{0} \end{array}\right.,\;$ $\begin{array}{l} \tau_{\max}=1.8\beta_{w}f_{\mathrm{ct}},{\;s}_{0}=2.5\tau_{\max}\left(\frac{t_{a}}{E_{a}}+\frac{50}{E_{c}}\right),\\ s_{f}=0.33\beta_{w},\;\beta_{w}=\sqrt{1.125\frac{2-b_{f}/b}{1+b_{f}/400}} \end{array}$
Dai and Ueda [34]	$\tau =\left\{ \begin{array}{l} \tau_{\max}{\left(\frac{s}{s_{0}}\right)}^{0.575}\mathrm{if}\;s\leq s_{0}\\ \tau_{\max}e^{-\beta \left(s-s_{0}\right)}\;\mathrm{if}\;s>s_{0} \end{array}\right.,$ $\begin{array}{l} \tau_{\max}=\frac{-1.575{\alpha K}_{a}+\sqrt{2.481\alpha^{2}K_{a}^{2}+6.3{\alpha \beta }_{w}{}^{2}K_{a}G_{f}}}{2\beta },{\;s}_{0}=\frac{\tau_{\max}}{{\alpha K}_{a}},\\ G_{f}=7.554K_{a}^{-0.449}{\left(f_{c}^{\prime }\right)}^{0.343},\;\beta_{w}=0.0035K_{a}{{(E}_{f}t_{f}/1000)}^{0.34},\\ K_{a}=G_{a}{/t}_{a},\;\alpha =0.028{{(E}_{f}t_{f}/1000)}^{0.254} \end{array}$
Lu et al. [35]	$\tau =\left\{ \begin{array}{l} \tau_{\max}\sqrt{\frac{s}{s_{0}}}\;\mathrm{if}\;s\leq s_{0}\\ \tau_{\max}e^{-\alpha \left(\frac{s}{s_{0}}-1\right)}\;\mathrm{if}\;s>s_{0} \end{array}\right.,\;$ $\begin{array}{l} \;\alpha ={\left(\frac{G_{f}}{\tau_{\max}s_{0}}-\frac{2}{3}\right)}^{-1},\tau_{\max}=1{.5\beta }_{w}f_{\mathrm{ct}},\;\\ G_{f}=0.308\beta_{w}^{2}\sqrt{f_{\mathrm{ct}}},\;\beta_{w}=\sqrt{\frac{2.25-b_{f}/b}{1.25+b_{f}/b}} \end{array}$
Obaidat et al. [36]	$K_{0}=0.16\frac{G_{a}}{t_{a}}{,\;\tau }_{\max}=1.46G_{a}^{0.165}f_{\mathrm{ct}}^{1.033},G_{f}=0{.52f}_{\mathrm{ct}}^{0.26}G_{a}^{-0.23}$

**Table 6:** Calculation of characteristic parameters of the traction–separation law.

Constitutive Models	K_0_ (MPa/mm)	τ_max_ (MPa)	G_f_ (N/mm)
Neubauer and Rostasy [31]	28.69	3.41	0.20
Nakaba et al. [32]	100.73	6.55	0.94
Monti et al. [33]	157.03	2.24	0.14
Dai and Ueda [34]	83.90	7.10	0.90
Lu et al. [35]	76.92	3.60	0.31
Obaidat et al. [36]	224.47	17.26	0.12

**Table 7:** Predictions of slabs’ loading-carrying capacity and corresponding mid-span deflection compared with experimental results.

Slab	Experiment		Prediction		$\frac{P_{u,\mathrm{theo}.}}{P_{u,\exp.}}$	$\frac{u_{\mathrm{theo}.}}{u_{\exp.}}$
	$P_{u,\exp.}\left(\mathrm{kN}\right)$	$\Delta_{u,\exp.}\left(\mathrm{mm}\right)$	$P_{u,\mathrm{theo}.}\left(\mathrm{kN}\right)$	$\Delta_{u,\mathrm{theo}.}\left(\mathrm{mm}\right)$		
Reference slab	43.5	18.5	39.9	16.8	0.92	0.91
Retrofitted slab	69.2	14.0	63.0	12.3	0.91	0.88

**Table 8:** FEM predicted results for retrofitted slab compared with experimental data.

Constitutive Models	P_u,mod._ (kN)	Δ_u,mod._ (mm)	K _mod._ (kN/mm)	$\frac{P_{u,\mathrm{mod}.}}{P_{u,\exp.}}$	$\frac{\Delta_{u,\mathrm{mod}.}}{\Delta_{u,\exp.}}$	$\frac{K_{\mathrm{mod}.}}{K_{\exp.}}$
Perfect bond	73.7	11.53	29.7	1.07	0.82	1.39
Neubauer and Rostasy [31]	69.8	10.83	28.1	1.01	0.77	1.32
Nakaba et al. [32]	73.6	11.57	29.2	1.06	0.83	1.37
Monti et al. [33]	72.2	10.77	29.4	1.04	0.77	1.38
Dai and Ueda [34]	73.6	11.58	29.1	1.06	0.83	1.37
Lu et al. [35]	70.4	10.81	29.0	1.02	0.77	1.36
Obaidat et al. [36]	72.6	10.71	29.5	1.05	0.77	1.38

## Data Availability

Not applicable.

## Nomenclature

A_s_	Tensile steel area	P_u,theo._	Prediction of carrying additional load capacity
b	Width of RC slab	K	Stiffness of slab
b_F_	Width of CFRP laminate	s	Local slip
c	Distance from extreme compression fiber to the neutral axis	s_f_	Maximum local slip
d	Distance from the extreme fiber of the compression zone to the center of the steel	s_0_	Local slip at τ_max_
E_a_	Elastic modulus of epoxy resin	t_a_	The thickness of the epoxy resin
E_c_	Elastic modulus of concrete	t_F_	The thickness of CFRP laminate
E_F_	Elastic modulus of CFRP	t_H_	The thickness of the concrete overlay
f′_c_	Compressive strength of concrete	j_1_d	Moment arm length from the center of concrete stress block to a steel
f_ct_	Tensile strength of concrete	j_2_d	Moment arm length from the center of concrete stress block to CFRP
f′_H_	Compressive strength of the concrete overlay	Δ_u_	Mid-span deflection
f_tF_	Tensile strength of CFRP	Δ_u,exp._	Mid-span deflection corresponds to experimental load-carrying capacity
G_a_	Shear modulus of epoxy resin	Δ_u,mod._	Mid-span deflection corresponds to modeling load-carrying capacity
G_c_	Shear modulus of concrete	Δ_u,theo._	Mid-span deflection corresponds to the predicted loading-carrying capacity
G_f_	Fracture energy	τ	Shear stress
G_n,_ G_s,_ G_t_	Fracture energies in the normal, first, and second directions of shear, respectively	τ_max_	Maximum shear stress
$\begin{array}{l} G_{n}^{f},{\;G}_{s}^{f},\\ G_{t}^{f} \end{array}$	Fracture energies to induce failure in the normal, first, and second directions of shear, respectively	$\begin{array}{l} \tau_{n},{\;\tau }_{s},\\ \tau_{t} \end{array}$	The normal, first, and second shear stress, respectively
h	Height of RC slab	σ_max_	Maximum nominal stress
K_a_	Shear stiffness of the epoxy resin	η	Parameter for cohesive material
K_c_	Shear stiffness of concrete	α	A parameter of bond–slip relationships
K_0_	Initial stiffness	β_w_	Width ratio factor
K_nn_, K_ss_, K_tt_	Elastic stiffnesses in the normal, first, and second directions of shear, respectively	γ_c_	Unit weight of concrete
P_u,exp._	Capacity for carrying additional loads in the experiment	〈 〉	Macaulay brackets, which implied that compressive stress does not lead to the initial damage
P_u,mod._	Capacity for carrying additional loads in the numerical modeling		
